# Supplementary material for: Sinorhizobium meliloti DnaJ Is Required for Surface Motility, Stress Tolerance, and for Efficient Nodulation and Symbiotic Nitrogen Fixation
Source: Int J Mol Sci. 2023 Mar 19;24(6):5848. doi: 10.3390/ijms24065848 (PMC10055731; doi:10.3390/ijms24065848)
Supplement: Supplementary file 1 [file ijms-24-05848-s001.zip › ijms-2266721-supplementary.pdf]

Supplementary Material

Sinorhizobium meliloti DnaJ Is Required for Surface Motility, Stress Tolerance, and for Efficient Nodulation and Symbiotic Nitrogen Fixation

Paula Brito-Santana [1], Julián J. Duque-Pedraza [1], Lydia M. Bernabéu-Roda [1], Cristina Carvia-Hermoso [1], Virginia Cuéllar [1], Francisco Fuentes-Romero [2], Sebastián Acosta-Jurado [3], José-María Vinardell [2] and María J. Soto [1,*]

List of Supplementary Material

Figure S1. Identification of flagellaless GR4flaAB-derivative transposants impaired in the response to volatile 2-tridecanone (2-TDC).

Figure S2. Multiple sequence alignment by MUSCLE of DnaJ amino acid sequences from different bacterial species

Figure S3. Growth of *S. meliloti dnaJ* mutants and their parental strains on solid and liquid media.

Figure S4. Effect of H_2O_2 on *S. meliloti* GR4 and GR4flaAB cell survival.

Figure S5. Appearance of alfalfa plants inoculated with *S. meliloti dnaJ* mutant strains at the end of the nodulation kinetics experiment.

Figure S6. Complementation of the symbiotic phenotype of *S. meliloti dnaJ* mutants.

Table S1. Bacterial strains and plasmids used in this study.

Table S2. List of primers used in this study

References

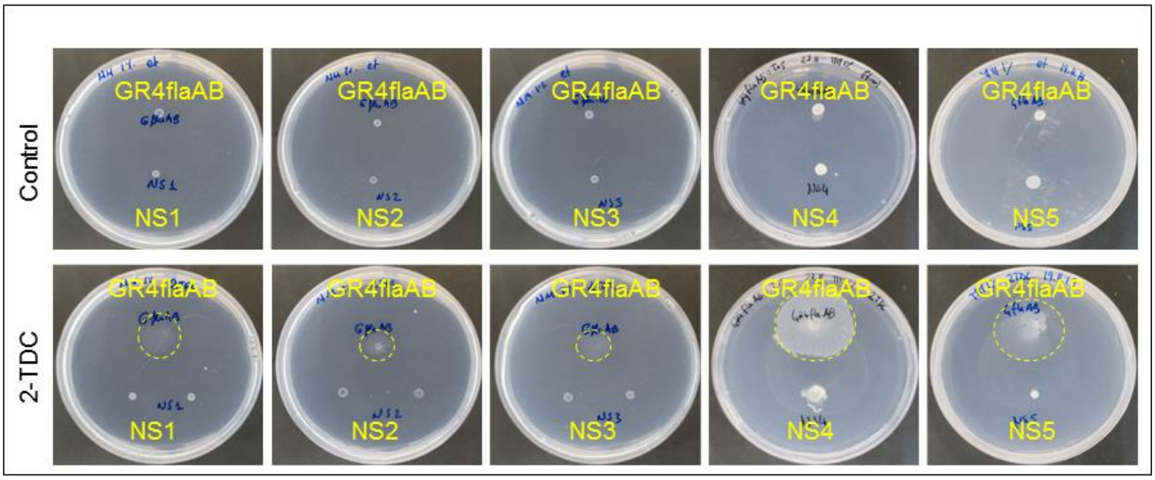

Figure S1. Identification of flagellaless GR4flaAB-derivative transposants impaired in the response to volatile 2-tridecanone (2-TDC). Surface motility assays on MM (1% agar) with *S. meliloti* GR4flaAB and GR4flaAB-derivative NS transposants in the presence of volatile 2-TDC. About 20 µl of either a solution containing 1 µmol 2-TDC, or ethanol (Control) were applied to the lid of the plates just before incubation. Representative pictures of the motilities exhibited after 48 h of incubation are shown.

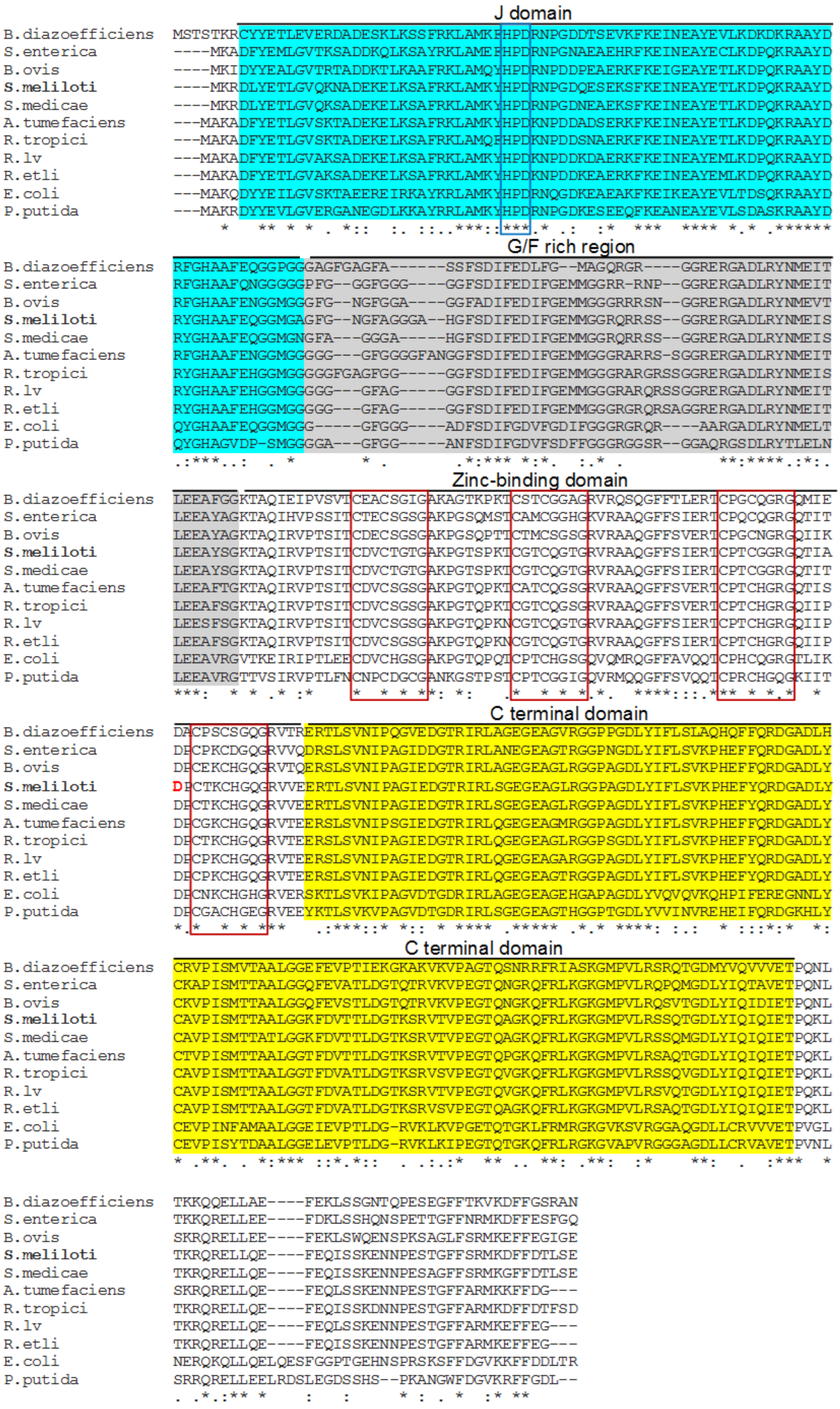

Figure S2. Multiple sequence alignment by MUSCLE of DnaJ amino acid sequences from different bacterial species. Identical residues are indicated with an asterisk. Colons and full stops indicate partial matches. Domains are highlighted in different colors following the color code used in Figure 1b of the

main manuscript. The evolutionarily conserved HPD motif of the J domain is indicated with a blue box. Conserved zinc binding motifs in the zinc-binding domain are indicated with reddish boxes. The insertion site for Tn*5* in the NS4 mutant at D_{201} is highlighted in red. The amino acid sequences are of the DnaJ proteins from *Bradyrhizobium diazoefficiens* USDA 110 (P94319), *Salmonella enterica* subsp. *enterica* serovar Enteritidis (MIL09225), *Brucella ovis* ATCC 25840 (Q05980), *S. meliloti* GR4 (AGA05171), *Sinorhizobium medicae* WSM419 (A6UEY1), *Agrobacterium tumefaciens* (*Rhizobium radiobacter*) RUOR (AAR84666), *Rhizobium tropici* CIAT899 (AGB69576), *Rhizobium leguminosarum* bv. *viciae* 3841 (Q1MN12), *Rhizobium etli* CFN42 (Q2KDW7), *Escherichia coli* K-12 (P08622), and *Pseudomonas putida* (*Arthrobacter siderocapsulatus*) PCL1445 (Q5BVD3).

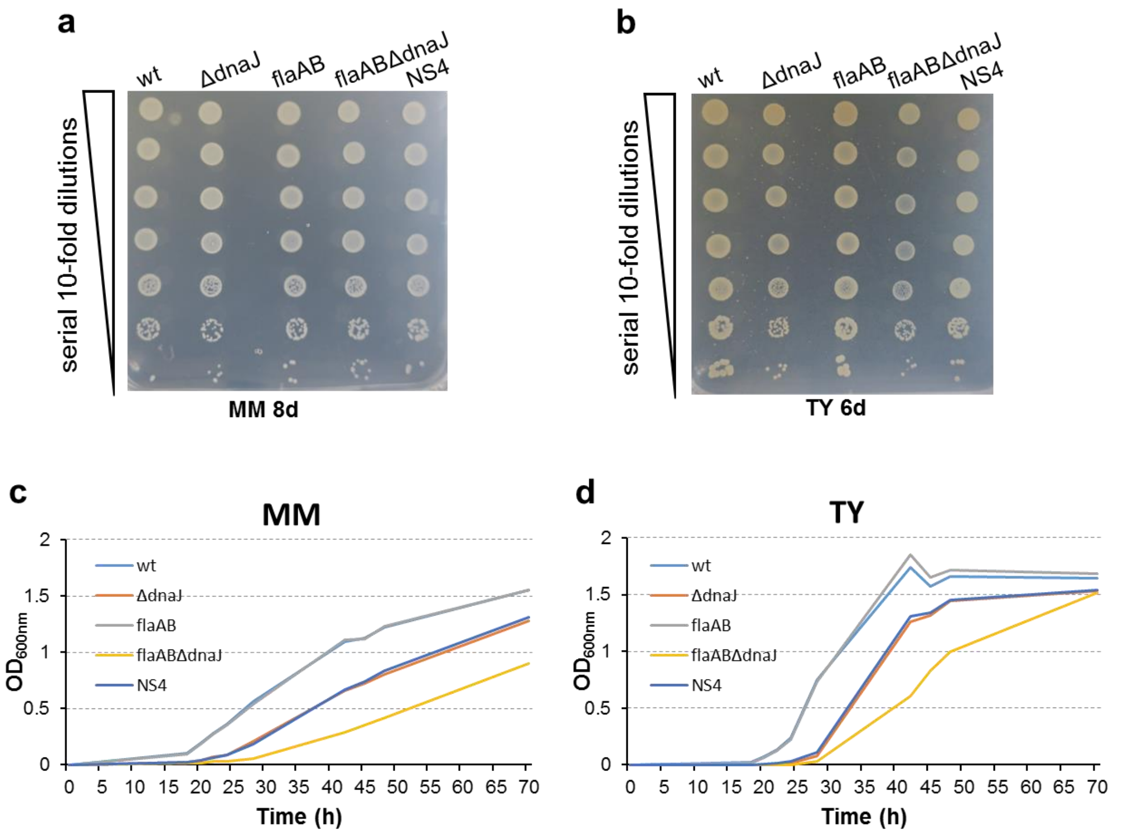

Figure S3. Growth of *S. meliloti dnaJ* mutants and their parental strains on solid and liquid media. Growth on (**a**) agar Minimal Medium (MM) and on (**b**) agar TY. Ten μl of 10-fold serial dilutions of mid exponential phase cultures of each strain were spotted on plates and incubated for 6 days (6d) or 8 days (8d) at 28ºC. Growth curves in liquid (**c**) MM and (**d**) TY were determined spectrophotometrically.

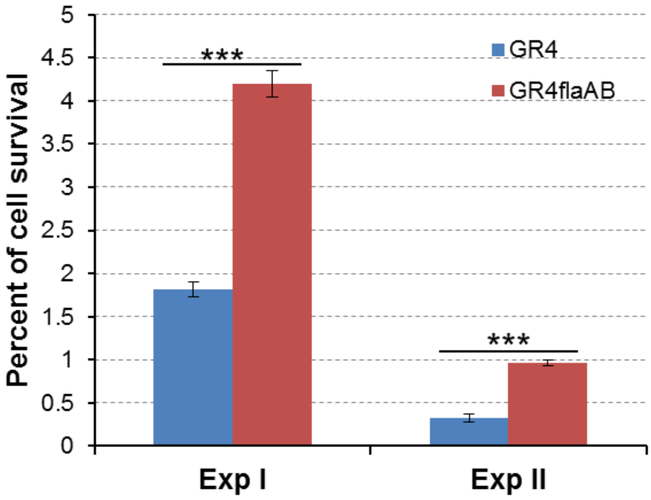

Figure S4. Effect of H_2O_2 on *S. meliloti* GR4 and GR4flaAB cell survival. Survival rates were determined after exposure to 1 mM H_2O_2 for 90 min. Error bars indicate standard errors. Asterisks indicate significant differences according to an ANOVA test ($p < 0.001$).

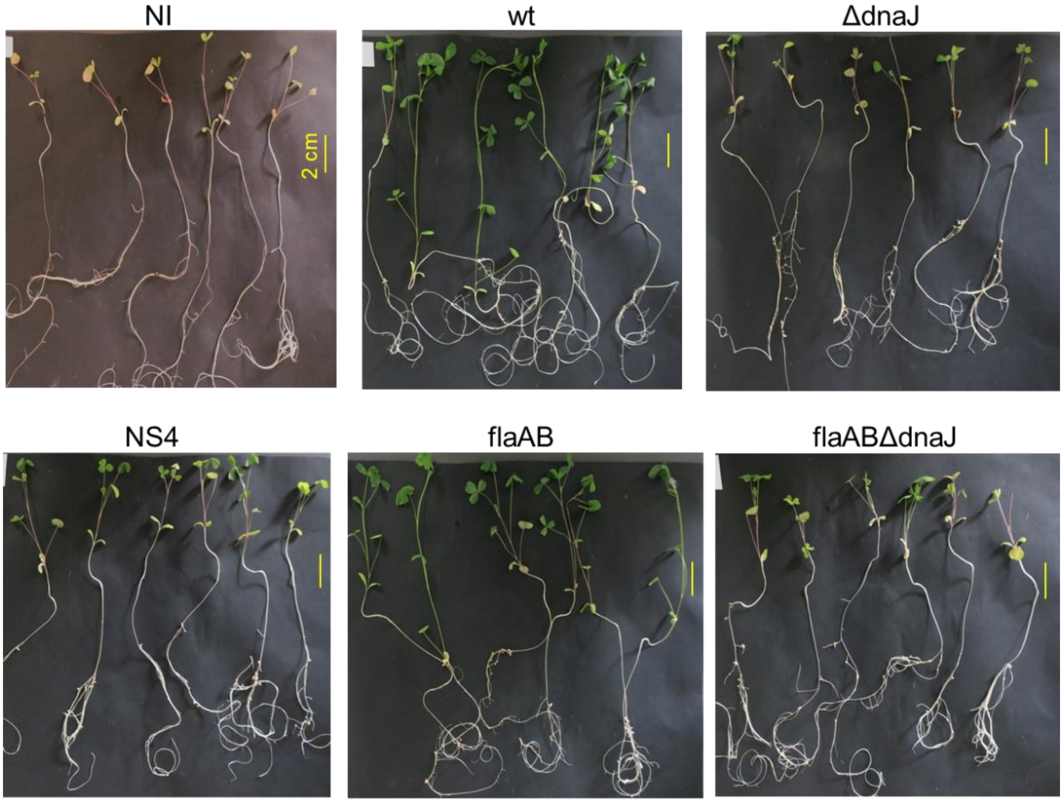

Figure S5. Appearance of alfalfa plants inoculated with *S. meliloti dnaJ* mutant strains at the end of the nodulation kinetics experiment. Alfalfa plants were grown in hydroponic culture under axenic conditions in glass tubes. Pictures were taken 21 days after inoculation with the rhizobial strains. The yellow bar represents 2 cm. NI, non-inoculated; wt, wild type strain GR4; ΔdnaJ, GΔdnaJ strain; flaAB, GR4flaAB strain.

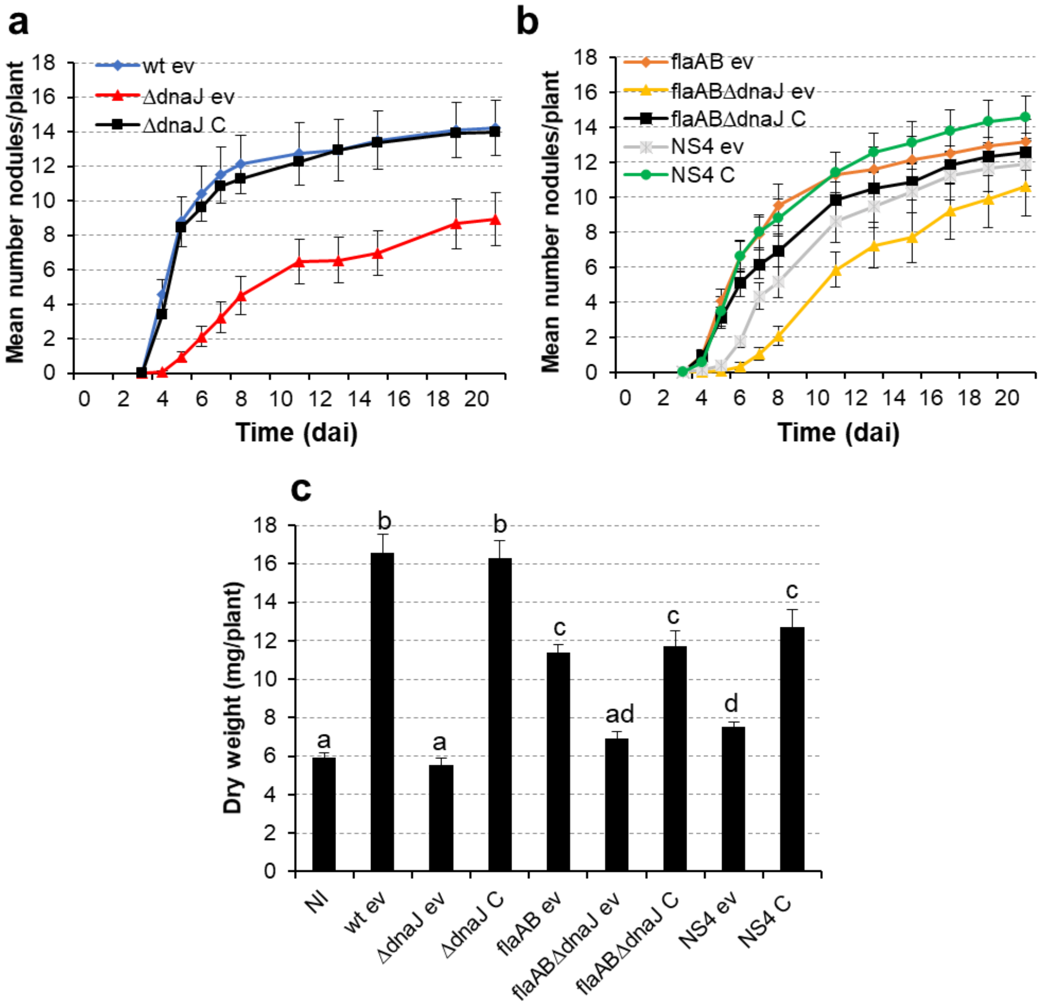

Figure S6. Complementation of the symbiotic phenotype of *S. meliloti dnaJ* mutants. Nodulation kinetics of (**a**) GR4 strain carrying the empty vector pJB3 (wt ev) and its derivative *dnaJ* deletion mutant carrying either pJB3 or the complementing plasmid pJ-dnaJ (ΔdnaJ ev and ΔdnaJ C, respectively), and (**b**) the flagellaless strain GR4flaAB carrying the empty vector (flaAB ev), and its *dnaJ* deletion (flaABΔdnaJ) and transposon insertion (NS4) derivative mutants carrying either the empty vector (ev) or the complementing plasmid (C). (**c**) Symbiotic efficiency of *S. meliloti* strains as determined by the shoot dry weight of alfalfa plants 21 days after inoculation (dai). NI, non-inoculated plants. Different letters indicate significant differences according to an analysis-of-variance (ANOVA) test (p≤0.05).

Table S1. Bacterial strains and plasmids used in this study.

Strain or plasmid	Relevant characteristics	Reference
S. meliloti		
GR4	Wild type strain	[1]
GR4flaAB	GR4 *flaAflaB*::Hyg, Hyg^r	[2]
NS1	GR4flaAB C770_GR4Chr0066::Tn*5*; Km^r, Hyg^r	This study
NS2	GR4flaAB C770_GR4Chr0263::Tn*5*; Km^r, Hyg^r	This study
NS3	GR4flaAB C770_GR4Chr1253::Tn*5*; Km^r, Hyg^r	This study
NS4	GR4flaAB *dnaJ*::Tn*5*; Km^r, Hyg^r	This study
NS5	GR4flaAB C770_GR4Chr3081::Tn*5*; Km^r, Hyg^r	This study
GΔdnaJ	GR4 derivative containing the deleted version of the *dnaJ* gene	This study
flaABΔdnaJ	GR4flaAB derivative containing the deleted version of the *dnaJ* gene	This study
E. coli		
DH5α	*supE44*, *DlacU169*, *f80*, *lacZDM*, *5hsdR171*, *recA1*, *endA1*, *gyrA96*, *thi-1*, *relA1*	Bethesda Research Lab
S17-1	*thi*, *pro*, *recA*, *hsdR*, *hsdM*, *RP4-2-Tc::Mu-Km::Tn7*	[3]
Plasmids		
pSUP2021	Plasmid for random mutageneis with Tn*5*; Ap^r, Tcr, Cm^r, Km^r	[3]
pCR2.1-TOPO	Cloning vector; Ap^r, Km^r	Invitrogen
pTOPO-dnaJ	pCR2.1-TOPO carrying the *S. meliloti* wild type *dnaJ*; Ap^r, Km^r	This study
pTOPO-ΔdnaJ	pCR2.1-TOPO carrying the deleted version of the *dnaJ* gene; Ap^r, Km^r	This study
pJB3Tc19	IncP cloning vector; Tc^r, Ap^r	[4]
pJ-dnaJ	pJB3Tc19 with the *S. meliloti dnaJ* gene cloned downstream from the *lacZ* promoter; Tc^r	This study
pK18*mobsacB*	Suicide plasmid; Km^r	[5]
pK18-ΔdnaJ	pK18*mobsacB* carrying the deleted version of the *dnaJ* gene; Km^r	This study

Table S2. List of primers used in this study.

Name	Sequence (5′-3′)[a]	Use
IS50(1)	CACGATGAAGAGCAGAAG	
IS50(2)	TAGGAGGTCACATGGAAGTCAGAT	Mapping Tn*5* insertions
ARB2	GGCCACGCGTCGACTAGTAC	
ARB6	GGCCACGCGTCGACTAGTACN_{10}CGCC	
dnaJ-F	TATAAGCTTCCTGTGGCAAGATAATCAGG (*Hin*dIII)	Construction of pJ-dnaJ
dnaJ-R	TTTAAGCTTTAATCGCCCAGAGCATTGGC (*Hin*dIII)	
dnaJ-1	TTTGAATTCGAGATCGAGAAGATGGTC (*Eco*RI)	
dnaJ-2	GGTGGTGACGTCGAACTTCGCGAGTTTGCGAAAGGC	Deletion of *dnaJ*
dnaJ-3	GCCTTTCGCAAACTCGCGAAGTTCGACGTCACCACC	
dnaJ-R	TTTAAGCTTTAATCGCCCAGAGCATTGGC (*Hin*dIII)	

[a] Restriction sites are underlined

References

1. Casadesús, J.; Olivares, J. Rough and fine linkage mapping of the *Rhizobium meliloti* chromosome. *Mol Gen Genet* **1979**, *174*, 203-209, DOI: 10.1007/BF00268356.
2. Bernabéu-Roda, L.; Calatrava-Morales, N.; Cuéllar, V.; Soto, M. Characterization of surface motility in *Sinorhizobium meliloti*: regulation and role in symbiosis. *Symbiosis* **2015**, 1-12, DOI: 10.1007/s13199-015-0340-4.
3. Simon, R.; Priefer, U.; Pühler, A. A broad host range mobilization system for *in vivo* genetic-engineering: transposon mutagenesis in Gram-negative bacteria. *Biotech* **1983**, *1*, 784-791, DOI: 10.1038/nbt1183-784.
4. Blatny, J.M.; Brautaset, T.; Winther-Larsen, H.C.; Haugan, K.; Valla, S. Construction and use of a versatile set of broad-host-range cloning and expression vectors based on the RK2 replicon. *Appl Environ Microbiol* **1997**, *63*, 370-379, DOI: 10.1128/aem.63.2.370-379.1997.
5. Schäfer, A.; Tauch, A.; Jager, W.; Kalinowski, J.; Thierbach, G.; Pühler, A. Small mobilizable multi-purpose cloning vectors derived from the *Escherichia coli* plasmids pK18 and pK19: selection of defined deletions in the chromosome of *Corynebacterium glutamicum*. *Gene* **1994**, *145*, 69-73, DOI: 10.1016/0378-1119(94)90324-7.
